# Efficacy of continuous ingestion of dewaxed brown rice on the cognitive functions of the residents of elderly welfare facilities: A pilot test using crossover trial

**DOI:** 10.1002/fsn3.1202

**Published:** 2019-09-27

**Authors:** Maya Uenobe, Toshiyuki Saika, Natsumi Waku, Masae Ohno, Hiroyuki Inagawa

**Affiliations:** ^1^ Department of Food and Health Sciences Faculty of Health and Human Development The University of Nagano Nagano Japan; ^2^ Course of Nutrition Management Graduate School of Human Life Science Nagoya University of Economics Aich Japan; ^3^ Toyo Rice Corporation Wakayama Japan; ^4^ Social Welfare Corporation Tanpopo Welfare Association TSURU no Sato Special Nursing Home for the Elderly Aich Japan; ^5^ Social Welfare Corporation Nishikasugai Welfare Association Heiann no Sato Special Nursing Home for the Elderly Aich Japan; ^6^ Research Institute for Healthy Living Niigata University of Pharmacy and Applied Life Sciences Niigata Japan; ^7^ Control of Innate Immunity, Technology Research Association Kagawa Japan

**Keywords:** brown rice, cognitive function, dewaxed brown rice, elderly, lipopolysaccharide

## Abstract

Compared with regular brown rice, dewaxed brown rice (DBR), prepared by excluding only the wax layer in the outermost layer of brown rice using a new rice milling technique, has improved water absorbency, digestibility, and taste. Dewaxed brown rice has a nutritional value close to that of brown rice and contains a large amount of lipopolysaccharides (LPS), which are known to improve the cognitive function of mice. In this study, we examined the effect of continuous DBR ingestion on the cognitive function of elderly people. A crossover comparison test was performed, in which elderly people who moved into an elderly welfare facility were divided into two groups and ingested DBR or polished white rice for three meals a day for 6 months, followed by a change in test meals for the next 6 months. Cognitive function was assessed using Revised Hasegawa's Dementia Scale (HDS‐R) before starting the test and 6 months after ingesting each test meal. No subjects withdrew or discontinued DBR intake during the study period, and all subjects continued the test for 6 months. In subjects with low cognitive function (defined as subjects with HDS‐R total score of ≥1 but <10 at the start of the study), there was a significant association between continuous DBR ingestion and cognitive function improvement (increase in total HDS‐R score). Our findings suggest that the long‐term DBR ingestion as a staple food could be useful for preventing cognitive decline in elderly; it offers an easily implemented option as a daily diet for preventing cognitive decline.

## 1 INTRODUCTION

Although a growing number of people are diagnosed with dementia worldwide, few effective treatments and drugs are available for dementia at present, which are projected to create a substantial economic and care burden on the society in the future (Canevelli, Lucchini, Quarata, Bruno, & Cesari, 2016; Prince, Comas‐Herrera, Knapp, Guerchet, & Karagiannidou, 2016). Reportedly, one of the most effective strategies for dementia prevention is nutritional intervention (Rakesh, Szabo, Alexopoulos, & Zannas, 2017). Indeed, several investigations have established a correlation between dietary patterns and cognitive functions (Morris et al., 2015, 2016; Tangney, 2015). Rice, which is mostly consumed as a staple food, is considered helpful in this regard. Lately, brown rice (BR), which is rich in nutrition and effective in promoting health maintenance across all its varieties, has garnered considerable attention (Sharif, Butt, Anjum, & Khan, 2014). It is considered that better nutrition is provided by BR than by polished rice, in which the bran layer and germ that contain vitamins E and B1, dietary fiber, and maltooligosaccharides are removed (Sharif et al., 2014).

Regarding the prophylactic effect of BR on dementia, experiments on mice have established that ferulic acid can activate mitochondria in the brain cells, potentially preventing Alzheimer's disease (AD) (Hagl et al., 2016; Okuda et al., 2018; Yan et al., 2001). However, BR is poor in taste because wax in the outermost (wax bran) layer inhibits water absorption, requiring a long immersion time, especially in case of inadequate water supply, thereby rendering it marginally acceptable (Pletsch & Hamaker, 2017). Notably, it is unsuitable for older adults with dementia as well as for those with decreased chewing or swallowing capabilities.

A recently developed type of BR called dewaxed brown rice (DBR), from which only the outermost wax bran layer is removed using a new rice milling technology, can potentially solve the abovementioned problems (Saika & Watanabe, 2017). Furthermore, DBR can be cooked as easily as polished white rice (WR) and can be added to any diet in the form of porridge or rice meal.

Previously, we have demonstrated that lipopolysaccharides (LPS) in DBR can activate macrophages primarily through the TLR4 pathway and, to a lesser extent, through the TLR2 pathway (Inagawa et al., 2016). Lipopolysaccharides is the primary component of the outer membrane of gram‐negative bacteria (e.g., *Acetobacter aceti*, *Zymomonas mobilis*, and *Xantomonas campestris*), which are symbionts of edible plants, such as cereals, vegetables, rice, wheat, and soybean. Reportedly, LPS extracted from plants activate macrophages (or immune cells), and oral LPS ingestion produces various physiologically active effects (Amano et al., 2015; Komori et al., 2015; Tamura, Inagawa, Nakata, Kohchi, & Soma, 2015).

Experiments using SAM‐P8 mice have revealed that orally administered LPS activates brain microglia, enhances amyloid β phagocytic ability as a cause of AD, and markedly decreases memory impairment (Kobayashi et al., 2017, 2018). Thus, continuous ingestion of LPS‐containing DBR as a staple food may be useful in slowing or even preventing the deterioration of cognitive function. However, the effects of LPS‐containing DBR on the cognitive functions of the elderly remain unclear. Hence, this study aimed to investigate the effects of continuous ingestion of DBR as a staple food on the cognitive functions of elderly people.

## 2 MATERIALS AND METHODS

### 2.1 Subjects

We enrolled 31 elderly participants (age, ≥65 years; range, 84.4 ± 9.5 years; seven males and 24 females) living in special nursing homes for the elderly. This study protocol was approved by the Ethics Committee of Nagoya Keizai University, Aichi, Japan (Receipt No. 2016‐1). Furthermore, we obtained written informed consent from each participant or their family members before their enrollment in this study.

### 2.2 Study design

**Figure 1:**
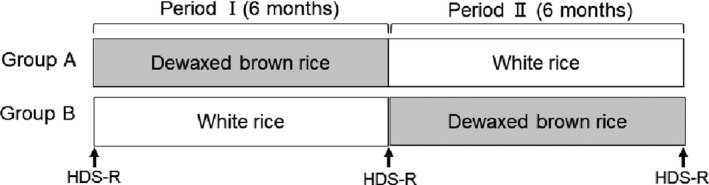
Study design. We conducted a controlled crossover trial in 31 elderly individuals in special nursing homes for the elderly; they were divided into two groups and ingested either dewaxed brown rice (DBR) or white rice (WR) three times a day for 6 months, followed by the ingestion of the other test food for the next 6 months. Cognitive function was assessed using the Revised Hasegawa dementia scale (HDS‐R) once every 6 months

We conducted a controlled crossover trial in special nursing homes for the elderly from August 2016 to July 2017. The test schedule is summarized in Figure 1. All participants were sorted into two groups (A and B)—those who ingested either DBR or WR three times a day for 6 months. During the WR intake period, regular meals were offered without changing the meal service of the facility; during the DBR intake period, no change was made to the dining plan of the facility, such as main dish, side dishes, and juice, and only DBR replaced WR as the staple food.

### 2.3 Dietary intervention

In this study, Koshihikari from Aichi Prefecture was used as WR. Koshihikari from Niigata Prefecture was used as raw material to produce DBR, which was process by Toyo Rice Corp. Regarding test meals, rice cooker RC‐18FE (TOSHIBA) was used for cooking regular rice meal, whereas rice cooker RC‐18 VSE (TOSHIBA) was used for cooking rice porridge. Test meals were prepared as per the manual developed by the facility staff in each nursing home, taking into account the eating function and everyday diets of their residents. In this context, the preparation of DBR required 2–3 parts water added to one part of DBR weight (g), and the rice porridge was cooked using five parts water added to one part rice. In addition, soft rice, which is a meal form between regular rice meal and rice porridge, was mixed at a 1:1 ratio of regular meal and rice porridge. Table 1 presents the principal nutritional values and the LPS contents of WR and DBR. We calculated the nutritional value of WR using the Japanese Food Standard Component Table 2017 (Seven Correction). Furthermore, the nutritional value of DBR was measured by the Japan Food Industry Association, and LPS content was measured by Macrophi Inc.

**Table 1:** Nutritive and LPS values of WR and DBR diets

Nutrients	WR	DBR
Calorie (kcal)	4	4
Protein (g)	0.6	0.5
Fat (g)	0.1	0.2
Carbohydrate (g)	9.4	6.5
Dietary fiber (g)	0.1	0.4
LPS (ng)	0.04	0.81

Note

Component values are indicated per 1 g dry weight.

Abbreviations: DBR, dewaxed brown rice; LPS, lipopolysaccharide; WR, white rice.

### 2.4 Eating conditions

In this study, eating conditions were visually inspected and recorded by the facility staff daily at all three meals, that is, breakfast, lunch, and dinner. Meals were primarily categorized into staple foods and side dishes, and scored from 0 to 10, as defined below, with a 10 indicating consumption of all the food provided and 0 if none was eaten.

### 2.5 Cognitive function

Revised Hasegawa's Dementia Scale (HDS‐R) is a test developed to screen for age‐associated dementia (Imai & Hasegawa, 1994), and it comprises nine simple items, which measure orientation, memory, attention/calculation, and fluency of words, with a maximum total score of 30 points. We used the HDS‐R (Imai & Hasegawa, 1994) to assess cognitive function. It was administered just prior to the beginning of the study and 6 months after each test meal intake.

### 2.6 Statistical analysis

In this study, data were analyzed using SPSS version 24.0 (IBM Corporation) for Windows. The values of participants' characteristics and eating conditions are presented as means and standard deviations. Revised Hasegawa's Dementia Scale scores are presented as means and standard errors. The normality of the data was confirmed using Kolmogorov–Smirnov test, and baseline characteristics of the population were compared using Student's *t* test. In this study, subjects were categorized into two groups: low cognitive function and high cognitive function. The former includes subjects with an HDS‐R total score of 1 or more and <10, whereas the latter includes subjects with HDS‐R total scores of 10 or more. The baseline of HDS‐R scores of WR and DBR intake was set at the beginning of each test meal intake in both groups. The total score of HDS‐R after ingestion of WR and DBR was set after intake of each test meal for 6 months in both groups. Additionally, “improvement” and “no improvement” of cognitive function are defined as follows: increase in the HDS‐R score compared with that at baseline was considered as “improvement,” and decrease or no change in the HDS‐R score was considered as “no improvement.” For categorical variables of both groups, two‐tailed Fisher's exact test was used to assess the independence of cognitive function. For each type of dietary intervention, WR and DBR groups were reorganized into two groups of 31 cases, respectively. A low cognitive function group also reorganized into 13 cases. Paired *t* test was used to assess the statistical significance of HDS‐R scores and differences between the paired changes in the two groups. Revised Hasegawa's Dementia Scale scores after ingestion of WR and DBR were analyzed using repeated measure analysis of variance, followed by post hoc analysis using Tukey's HSD test. A *p*‐value of <.05 was considered significant for all tests.

## 3 RESULTS

### 3.1 Diet parameters

Total calorie consumption was the same for DBR and WR. Protein (g) contents were 0.5 and 0.6 and fat (g) contents were 0.2 and 0.1 for DBR and WR, respectively. Carbohydrates (g) contents were 6.5 and 9.4 and dietary fiber (g) contents were 0.4 and 0.1 for DBR and WR, respectively. Lipopolysaccharides (ng) contents in the DBR and WR diets were 0.81 and 0.04, respectively (Table 1).

### 3.2 Participants' characteristics

Table 2 summarizes the characteristics of the participants, where 18 were in group A and 13 in group B. It turned out that there were no significant differences in the characteristics of these two cohorts. Specifically, the group A subjects had an average age of 84.3 ± 10.3 years, a body weight of 44.5 ± 10.0 kg, while their average body mass index (BMI) was 19.0 ± 5.5 kg/m^2^, their nursing care level averaged 3.4 ± 1.0, and their average score on the HDS‐R was 7.1 ± 10.0. In not much contrast, group B had an average age was 83.8 ± 9.1 years, body weight of 47.3 ± 8.9 kg, an overall BMI of 18.8 ± 7.0 kg/m^2^, and their nursing care level was 3.8 ± 0.7, while their HDS‐R was 7.1 ± 10.0. Regarding the dietary patterns, group A had eight people on regular meals, and 10 ate rice porridge. Group B had five people eating regular meals, with six preferring soft rice, and two specialized in eating rice porridge.

**Table 2:** Participants' characteristics at the start of the test

Variable		Group A	Group B	*p*
Number (men/women)		18 (4/14)	13 (3/10)	–
Age (years)		84.3 ± 10.3	83.8 ± 9.1	.768
Body weight (kg)		44.5 ± 10.0	47.3 ± 8.9	.428
BMI (kg/m^2^)		19.0 ± 5.5	18.8 ± 7.0	.922
Nursing care level		3.4 ± 1.0	3.8 ± 0.7	.178
HDS‐R		7.1 ± 10.0	8.0 ± 6.9	.785
Form of meals (*n*)	Regular meal	8	5	–
	Soft rice	–	6	–
	Rice porridge	10	2	–

Note

Data are presented as mean and standard deviation, and *p* values obtained by Student's *t* test, not significant. Nursing care level, between 1 and 5, based on the assessment of care requirements.

Abbreviations: BMI, body mass index; HDS‐R, Revised Hasegawa Dementia Scale.

### 3.3 Eating conditions

For both the staple food and the side dishes, the eating conditions were evaluated by an “eating score,” the details of which are shown in the Materials and Methods section. The average eating score of all participants during the 3 months before the test began was 9 ± 1. During the test period, the eating score regarding staple food was also 9 ± 1 and for the side dishes it was 9 ± 2, during both the WR and DBR ingestion segments, as detailed in Table 3. There are no statistically significant differences between these two groups regarding these various eating behaviors.

**Table 3:** Eating conditions in the period of ingesting WR and DBR shown as eating score

	Staple food	Others (main and side dishes)
Three months before the start of the test	9 ± 1	9 ± 1
Period of WR intake	9 ± 1	9 ± 2
Period of DBR intake	9 ± 1	9 ± 2

Note

Data are presented as mean and standard deviation. Student's *t* test, not significant. Meals were primarily categorized into staple foods and others (main dishes and side dishes) and scored from 0 to 10 (eating score was defined as below 10 when finished and 0 when all was left uneaten).

Abbreviations: DBR, dewaxed brown rice; WR, white rice.

### 3.4 Cognitive function using the HDS‐R

The total score of HDS‐R at the start of the test and after ingestion of WR and DBR in all subjects is presented in Table 4. The total score of HDS‐R in all subjects was 7.5 ± 1.6 at the start of the test, 8.7 ± 1.4 at WR, and 8.3 ± 1.4 after DBR ingestion. There was no significant difference in the total score of HDS‐R between the start of the test and after WR and DBR ingestion in all subjects, respectively. Table 5 shows the difference of the total score of each subject HDS‐R in each group. In group B, the difference in score was −1.0 ± 0.8 in WR, whereas it was 1.3 ± 0.9 after DBR ingestion. However, there was no significant difference in the total score between WR and DBR in group A, group B, and group A + B. Table 6 shows the difference in HDS‐R total score in the low cognitive function group (HDS‐R total score of 1 or more and <10). It was −0.5 ± 0.5 for ingestion of WR and 2.8 ± 0.7 for ingestion of DBR. In this group, the total score of HDS‐R significantly increased with ingestion of DBR compared with WR (paired *t* test, *p* = .001). Next, we investigated the number of subjects whose score increased (improvement) and decreased/did not change (no improvement) compared with the HDS‐R total score at baseline in the low cognitive function group (Table 7). We found that “improvement” was 3 in WR and 10 in DBR, whereas “no improvement” was 10 in WR and 3 in DBR. Subjects ingesting DBR had significantly more “improvement” than those ingesting WR (Fisher's exact test, *p* = .017). Table 8 shows the difference in score of each item of HDS‐R in the low cognitive function group. There was no item of HDS‐R with a significant difference between WR and DBR. Although statistically significant differences were not observed, visual memory as the eighth item was 0.1 ± 0.3 in WR and 1.3 ± 0.7 in DBR (paired *t* test, *p* = .136).

**Table 4:** Total score of HDS‐R at the start of the test and after ingestion of WR and DR

	Total score of HDS‐R	*p*
At the start of the test	7.5 ± 1.6	.227†
WR	8.7 ± 1.4	.844‡
DBR	8.3 ± 1.4	.512§

Note

Data are presented as mean and standard error, and *p* values obtained by repeated measure analysis of variance, followed by Tukey's HSD test.

Abbreviations: DBR, dewaxed brown rice; WR, white rice.

^†^ *p* = .227, between at the start of the test and WR.

^‡^ *p* = .884, between WR and DBR.

^§^ *p* = .512, between test start and DB. *n* = 31.

**Table 5:** Difference of total score of each subject HDS‐R in each group

	Difference in total score of HDS‐R		*p*
	WR	DBR	
Group A	1.6 ± 0.7	1.1 ± 0.9	.683
Group B	−1.0 ± 0.8	1.3 ± 0.9	.063
Group A + B	0.5 ± 0.6	1.2 ± 0.6	.441

Note

Data are presented as mean and standard error, and *p* values obtained by paired *t* test. Group A, *n* = 18; Group B, *n* = 13; and Group A + B, *n* = 31.

Abbreviations: DBR, dewaxed brown rice; WR, white rice.

**Table 6:** Difference of total score of HDS‐R in low cognitive function group

	Difference in total score of HDS‐R	*p*
WR	−0.5 ± 0.5	.001
DBR	2.8 ± 0.7	

Note

Data are presented as mean and standard error, and *p* values obtained by paired *t* test. A low cognitive function group includes the subjects with an HDS‐R total score of ≥1 and <10. *n* = 13.

Abbreviations: DBR, dewaxed brown rice; WR, white rice.

**Table 7:** Cross tabulations on “Improvement” and “No improvement” of cognitive function after ingestion of WR and BR in low cognitive function group

	Improvement	No improvement	*p*
WR	3	10	.017
DBR	10	3	

Note

The values in the table indicate the number of people. Improvement, Increase in HDS‐R score compared with baseline; No‐improvement, Decrease or no change in HDS‐R score. *p* values obtained by Fisher's exact test, two‐sided test. Low cognitive function group included the subjects with an HDS‐R total score of ≥1 and <10. *n* = 13.

Abbreviations: DBR, dewaxed brown rice; WR, white rice.

**Table 8:** Difference of the score of each item of HDS‐R in the low cognitive function group

Item of HDS‐R (Questions)	Allotment of scores	WR	DBR	*p*
1. Age (self‐orientation)	1	−0.2 ± 0.1	0.0 ± 0.2	.502
2. Date (date‐orientation)	4	0.0 ± 0.0	0.0 ± 0.1	1.000
3. Place (place‐orientation)	2	−0.2 ± 0.2	0.2 ± 0.1	.240
4. Repeating of three words† (working memory)	3	−0.2 ± 0.4	0.8 ± 0.5	.245
5. Serial subtractions of 7s (calculation)	2	0.2 ± 0.3	0.0 ± 0.3	.805
6. Digits backward (working memory)	2	0.1 ± 0.1	0.0 ± 0.2	.673
7. Recalling of three words† (short‐term memory)	6	−0.2 ± 0.1	0.7 ± 0.6	.132
8. Recalling five objects‡ (visual memory)	5	0.1 ± 0.3	1.3 ± 0.7	.136
9. Word fluency (fluency)	5	0.2 ± 0.2	0.8 ± 0.7	.485

Note

Data are presented as mean and standard error, and *p* values obtained by paired *t* test. Low cognitive function group included the subjects with an HDS‐R total score of ≥1 and <10. *n* = 13.

Abbreviations: DBR, dewaxed brown rice; WR, white rice.

^†^ For example, cherry blossom, cat and train, or plum, dog, and car.

^‡^ For example, watch, key, cigarette, pen, and coin.

## 4 DISCUSSION

In this pilot test, the effect of DBR compared with WR on cognitive function of the residents of elderly welfare facilities was investigated in 6‐month crossover trial. We hypothesized that LPS, the nutrient‐rich functional component of DBR, can be useful as a staple food to maintain homeostasis in the brain. Brown rice consumption in regular meals is highly recommended to derive benefits from the functional ingredients of rice. However, BR is not extensively consumed as a daily staple food because of its poor digestibility and taste. In particular, elderly people with poor chewing or swallowing capabilities find it difficult to eat. Of note, our findings are the first to demonstrate that DBR could be ingested over prolonged periods even by the elderly with reduced ability to chew or swallow. Because DBR overcomes the disadvantages of conventional BR, it can become a staple food useful for supporting the maintenance of brain health for elderly.

Previously, we have reported that because DBR contains bran and germ that are rich in LPS as a functional ingredient, its ability to activate macrophages is nearly 100 times higher compared with WR (Inagawa et al., 2016). Lipopolysaccharides present in the outer membrane of gram‐negative bacteria regulates the innate immunity by activating macrophages (Abreu, 2010). Lipopolysaccharides is known as an endotoxin that causes serious inflammation when it is released in the blood during the infection of gram‐negative bacteria (Schryvers, Schollaardt, Woods, Williams, & Bryan, 1987). However, LPS is a substance that is universally present in the environment by attaching to edible plants or floating in the air. It is reported that orally and transdermally ingested LPS is not toxic, and LPS contributes to the activation of innate immunity (Gen‐Ichiro et al., 2008; Oketani, Inoue, & Murakami, 2001; Schryvers et al., 1987). Plants such as cereals contain LPS derived from symbiotic gram‐negative bacteria. For example, Pantoea agglomerans, a gram‐negative bacterium, symbiotically grows with various edible plants, such as wheat and rice (Lindh, Kjaeldgaard, Frederiksen, & Ursing, 1991). Recently, in vitro studies have reported that *Pantoea agglomerans* LPS (LPSp) markedly increases the phagocytic activity directed at Aβ1–42 peptides in a mouse microglial cell line as well as in primary microglia isolated from the mouse brain (Kobayashi et al., 2016, 2017). In addition, the oral administration of LPSp markedly prevented a memory deficit in high‐fat diet‐fed SAMP8 mice (Kobayashi et al., 2018). In addition, insoluble amyloid β level was reduced in the brains of LPSp‐ingesting mice. Our results suggest that mainly compared with the WR‐based diet, continued DBR intake resulted in more LPS intake, which may have contributed to the prevention of subject's cognitive decline. Comparing DBR and WR used in this study, the component with the largest difference was LPS. Lipopolysaccharides content of DBR was about 20 times higher than that of WR. However, the amount of LPS actually consumed by an individual should be different for regular meal, soft rice, and rice porridge. The inability to determine the amount of rice‐derived LPS consumed by individuals limits our research. Although the effect and extent of the effect of DBR‐derived LPS on cognitive function could not be proved in this study, DBR may contribute to the prevention of cognitive decline.

Also, rice bran is a rich natural source of antioxidant polyphenols, flavonoids, phytic acid, vitamin E, and γ‐oryzanol (Moure et al., 2001); these useful ingredients are lost in the rice milling process used to produce WR (Tuncel & Yılmaz, 2011). Among these components, γ‐oryzanol is a specific functional component that is present in a high concentration in rice bran. Rice bran extract as a rich source of γ‐oryzanol improved impaired mitochondrial function in a cell culture AD model (Hagl et al., 2015) and affected the activation of microglia in vitro (Bhatia, Baron, Hagl, Eckert, & Fiebich, 2016). Recent studies have reported that the oral administration of rice bran extract containing γ‐oryzanol alleviated brain mitochondrial dysfunction in NMRI mice (Hagl et al., 2016). Dewaxed brown rice is rich in these components because only the outermost wax layer is removed from the grains (Watanabe & Hirakawa, 2018). However, whether γ‐oryzanol is included in the DBR used in this study remains to be confirmed and is a topic for our future investigations. Although we hypothesize that LPS is a major functional component of DBR, the interaction between LPS and other functional components may also contribute to the prevention of cognitive decline.

Our study has several limitations. First, only 31 elderly participants were included in the trial. Therefore, it cannot eliminate the possibility that the sample size was small to reach statistical significance. Second, it was nearly impossible to evaluate by invasive methods, such as blood biochemistry of elderly with physical and cognitive decline who were admitted to the institution; therefore, we recognized that this is a limitation to the present study. Revised Hasegawa's Dementia Scale cannot evaluate visuospatial and language abilities. Although dementia index can assess a wide range of performance and biochemical tests increase the chance of observing the positive change in cognitive functions, we selected HDS‐R as the most appropriate dementia index for the subjects of this study. Thirdly, the content of the components in DBR considered to be related to the maintenance of brain health, such as ferulic acid contained in brown rice, was not measured. Although we hypothesized that LPS contained in DBR is one of the substances that play an important role in maintaining brain function, it should be noted that other components are also involved. By not measuring other components that affect cognitive function, some effects that contributed to the observed differences may have been missed.

In conclusion, the present findings suggest that the ingestion of DBR as a staple food may be effective in preventing cognitive decline, at least in elderly people with low cognitive function. Of note, our findings demonstrate that DBR could be ingested over prolonged periods even by the elderly with reduced ability to chew or swallow. Therefore, it offers an easily implemented option as a daily diet for the prevention of cognitive function in elderly.

## CONFLICT OF INTEREST

Toshiyuki Saika is an employee of Toyo Rice Corp. All other authors declared no competing interests.

## ETHICAL APPROVAL

This investigation was conducted according to the principles expressed in the Declaration of Helsinki. Authors confirm that all the experiments conducted in the present study comply with the current laws in Japan. All protocols and procedures were ethically reviewed and approved by the Ethic Committee of Nagoya University of Economics (Receipt No. 2016‐1). Written informed consent was obtained from all study participants.
